# Advances in mesenchymal stem cell conditioned medium-mediated periodontal tissue regeneration

**DOI:** 10.1186/s12967-021-03125-5

**Published:** 2021-11-04

**Authors:** Hongbing Lin, Huishan Chen, Xuetao Zhao, Zhen Chen, Peipei Zhang, Yue Tian, Yawei Wang, Tong Ding, Lijing Wang, Yuqin Shen

**Affiliations:** 1 Department of Periodontics, Affiliated Stomatology Hospital of Guangzhou Medical University, Guangzhou Key laboratory of Basic and Applied Research of Oral Regenerative Medicine, Guangzhou, Guangdong 510182 China; 2 Jilin Provincial Key Laboratory of Tooth Development and Bone Remodeling, Hospital of Stomatology, Jilin University, Changchun, Jilin 130021 People’s Republic of China; 3 Guangzhou Key Laboratory of Basic and Applied Research of Oral Regenerative Medicine, Affiliated Stomatology Hospital of Guangzhou Medical University, Guangzhou, China; 4 Vascular Biology Research Institute, School of Life Sciences and Biopharmaceutics, Guangdong Pharmaceutical University, Guangzhou, China

**Keywords:** Periodontal tissue regeneration, Mesenchymal stem cells conditioned medium, Osteogenesis, Immunomodulation, Angiogenesis, Chemotaxis

## Abstract

Periodontitis is a chronic inflammatory disease that leads to the destruction of both soft and hard periodontal tissues. Complete periodontal regeneration in clinics using the currently available treatment approaches is still a challenge. Mesenchymal stem cells (MSCs) have shown promising potential to regenerate periodontal tissue in various preclinical and clinical studies. The poor survival rate of MSCs during in vivo transplantation and host immunogenic reaction towards MSCs are the main drawbacks of direct use of MSCs in periodontal tissue regeneration. Autologous MSCs have limited sources and possess patient morbidity during harvesting. Direct use of allogenic MSCs could induce host immune reaction. Therefore, the MSC-based indirect treatment approach could be beneficial for periodontal regeneration in clinics. MSC culture conditioned medium (CM) contains secretomes that had shown immunomodulatory and tissue regenerative potential in pre-clinical and clinical studies. MSC-CM contains a cocktail of growth factors, cytokines, chemokines, enzymes, and exosomes, extracellular vesicles, etc. MSC-CM-based indirect treatment has the potential to eliminate the drawbacks of direct use of MSCs for periodontal tissue regeneration. MSC-CM holds the tremendous potential of bench-to-bed translation in periodontal regeneration applications. This review focuses on the accumulating evidence indicating the therapeutic potential of the MSC-CM in periodontal regeneration-related pre-clinical and clinical studies. Recent advances on MSC-CM-based periodontal regeneration, existing challenges, and prospects are well summarized as guidance to improve the effectiveness of MSC-CM on periodontal regeneration in clinics.

## Background

Periodontitis is a complicated chronic inflammatory oral disease, which is globally prevalent and has direct involvement of vast oral microbiome, oral tissues and immune cells [1, 2]. Periodontitis could cause irreversible destruction of periodontal tissues, including periodontal ligament (PDL), cementum, and alveolar bone [3]. Disrupted microbial homeostasis in oral cavity may increase the risk of occurrence of various systemic diseases, including colitis, myocardial infraction, and Alzheimer’s diseases [4–9]. Therefore, the effective treatment of periodontitis and periodontal regeneration is crucial for human health.

Periodontal tissue regeneration involves the regeneration of the gingiva, alveolar bone, PDL and cementum. Among them, the regeneration and natural alignment of PDL is so far one of the most challenging tasks in the field of tissue engineering [10]. To regenerate lost periodontal tissue, numerous procedures and products have been developed such as guided tissue regeneration (GTR), application of platelet-rich plasma, natural graft tissues and synthetic biomaterials [11–16]. However, most of the current or emerging paradigms have either proven to have limited and variable outcomes or have not been developed for clinical use [17].

Stem cell-based periodontal regeneration is currently at the center of attention [18]. Different cell types such as bone marrow MSCs (BMSCs), periodontal ligament stem cells (PDLSCs), dental pulp stem cells (DPSC) are key stem cells used in stem cell-based periodontal regeneration [19]. However, stem cell-based therapies have some serious limitations, including dedifferentiation during MSCs amplification, reduction of regeneration efficiency after administration, inconsistent quality control in large-scale cell production, and the invasive procedure of MSCs isolation [20–24]. In addition, it has been reported that in vivo monitoring of transplanted MSCs in an acute myocardial infarction tracked only 4.4% of MSCs in the transplanted site after 1 week, which indicated the poor survival rate of transplanted MSCs. Interestingly, the MSCs grafting promotes the functional improvement of the infarcted heart suggesting the role of MSCs released trophic factors on native cardiac and vascular cells’ function [25]. This suggests the role of stem cell-released signaling molecules and factors on tissue regeneration.

Shreds of evidence suggest that MSCs enhance immune responses during early-stage inflammation through the paracrine and autocrine manners, and subsequent tissue regeneration by producing a spectrum of protective bioactive factors [26, 27]. The factors are broadly defined as secretome or conditioned medium (CM), and usually classified as cytokines, chemokines, cell adhesion molecules, lipid mediators, interleukins, growth factors, hormones, exosomes, microvesicles, etc. [28]. The CM from stem cells can play a major role in tissue repair and regeneration [29]. As a cell-free technique, MSC-CM transplantation is more convenient and safer to apply and has greater potential for clinical translation than direct MSCs transplantation [30, 31]. MSC-CM provides several key advantages over cell-based applications: (a) MSC-CM employs the administration of proteins instead of whole cells that avoids the risk of host immune reactions; (b) MSC-CM can be stored for a relatively long period without any toxic cryopreservatives such as DMSO; (c) MSC-CM is cost-effective; (d) Evaluation of CM for safety and efficacy is much simpler compared to conventional pharmaceutical agents or MSCs [32]. Moreover, MSC-CM has immunomodulatory and tissue regenerative potential [33, 34]. Therefore, the use of MSC-CM could be an effective approach to regenerate periodontal tissue in the inflammatory environment of periodontitis. The therapeutic use of MSC-CM in periodontal regeneration is still a new frontier. The present review discusses the current understanding of the use of CM for periodontal tissues regeneration in preclinical and clinical studies, existing challenges, and prospects.

## Periodontal tissue regeneration

Periodontitis results from oral microbial dysbiosis, which disrupts the ecologically balanced biofilm associated with periodontal tissue homeostasis and finally causes destruction of the tooth-attachment apparatus, including gingiva, alveolar bone, root cementum, and PDL [6]. The dysbiotic microbes induce host immune response recruiting mucosal epithelial cells and gingival fibroblasts, and immune cells such as mononuclear phagocytes (MNPs), antigen-presenting cells (APCs), and specific T cell subsets (type 17 helper T cells, Th17 cells) in the periodontal region. The interaction between dysbiotic microbes and the host cells leads to the release of inflammatory cytokines [35]. The main components of these cytokines are interleukin-1 (IL-1) [36], IL-6 [37], and tumor necrosis factor (TNF) family [38]. These are key pro-inflammatory cytokines that promote tissue destruction. Secondly, cytokines secreted by MNPs, APCs, and local lymphocytes lead to the differentiation of a specific subset of inflammatory lymphocytes. The stimulation of IL-1 and IL-6 family cytokines induces osteoclast formation and activity in the bony niche [35].

The true regeneration of periodontium includes alveolar bone, PDL, and cementum, which is characterized by newly formed alveolar bone and cementum connected by regenerated periodontal ligament fibers aligned in certain direction [10]. It has been reported that the regeneration of periodontium may occur simultaneously, although the osteogenic process may be slightly prior to the differentiation of cementum and fibers [39]. Therefore, the structural and interactive complexity of periodontal tissue is the key challenge for effective and functional regeneration.

The purpose of periodontal therapy is to control the infection and reconstruct the structure and function of periodontal tissues. The effectiveness of traditional treatments in periodontal tissue regeneration is still limited and unpredictable [10]. Tissue engineering is a new cutting-edge technology which involves stem cells, cytokines, and scaffolds. In recent years, the application of tissue engineering in periodontal tissue regeneration is increasing [7, 16, 19], the regeneration of alveolar bone [40–43], PDL [44–46], cementum [47, 48] and even the entire bone-PDL-cementum complex [39] has gained success to some extent.

## Sources of MSCs

**Fig. 1:**
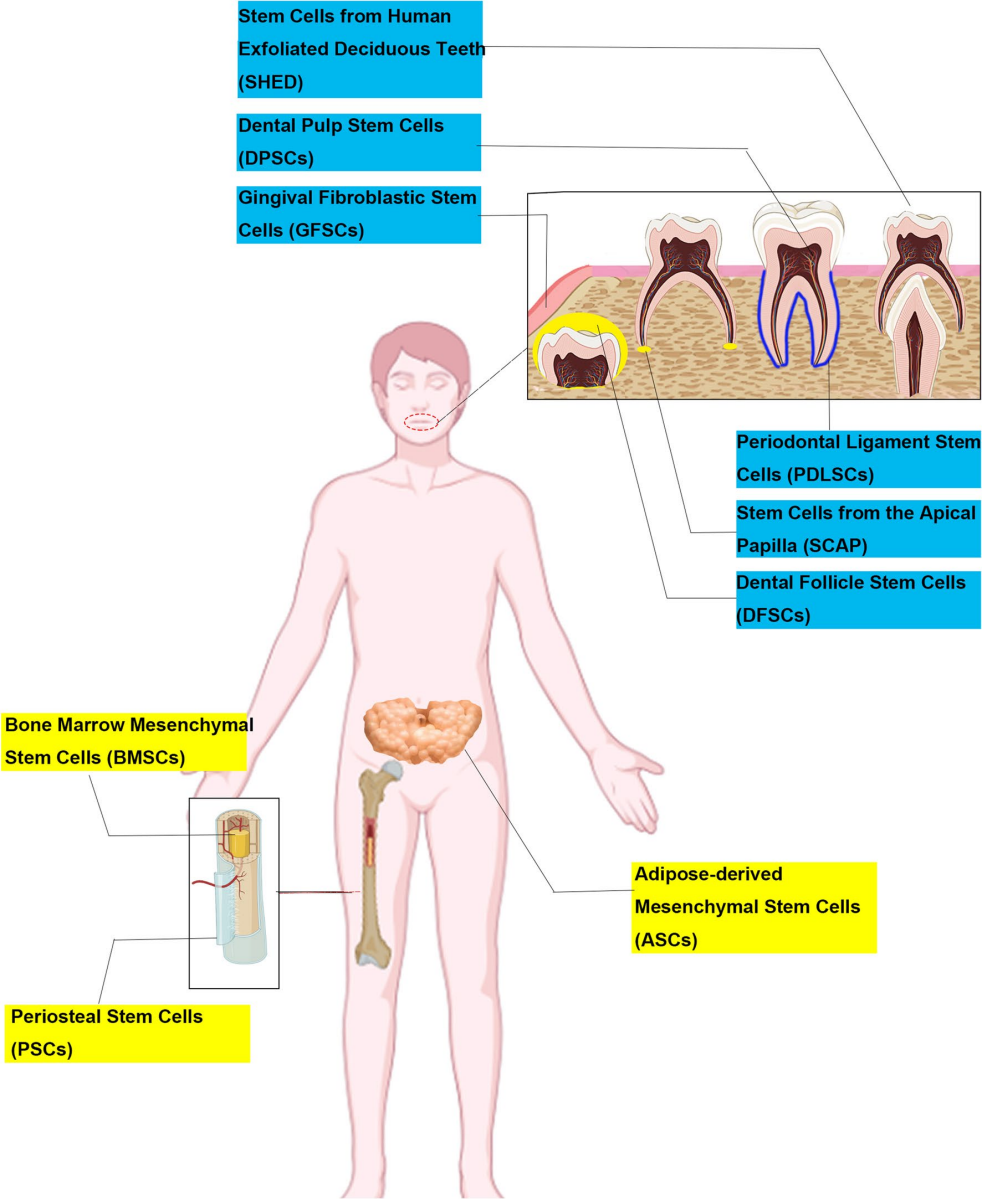
Sources of mesenchymal stem cells that are commonly used for tissue regeneration applications

Stem cells are at the forefront of new therapies because of their ability to self-renew and differentiate towards various cell lineages [49]. Stem cells are mainly composed of embryonic stem cells and somatic stem cells. Somatic stem cells include both hematopoietic stem cells (HSCs) and MSCs [50]. Mesenchymal and Tissue Stem Cell Committee of the International Society for Cellular Therapy have proposed the minimal criteria to define human MSCs. [1] MSCs are plastic-adherent when maintained in standard culture conditions. [2] MSCs express CD105, CD73 and CD90, and lack expression of CD45, CD34, CD14 or CD11b, CD79a or CD19 and HLA-DR surface molecules. [3] MSCs have osteogenic, adipogenic, and chondrogenic plasticity in vitro [51, 52]. A variety of studies have demonstrated that MSCs have great potential in bone and dental tissue regeneration. The most commonly used stem cells are BMSCs [53], periosteal stem cells (PSCs) [54], adipose-derived mesenchymal stem cells (ASCs) [55], and dental tissue-derived stem cells (DSCs) [56], which include PDLSCs [57], dental pulp stem cells (DPSCs) [58], gingival fibroblastic stem cells (GFSCs) [59], dental follicle stem cells (DFSCs) [60], stem cells from human exfoliated deciduous teeth (SHEDs) [61], and stem cells from the apical papilla (SCAP) [62] (Fig. 1). In addition, the tissues harvested during dental implant are also an important source of DSCs [63, 64].

MSCs from different sources display tissue reparative potential [65, 66]. MSCs have garnered significant interest in tissue engineering due to their immunomodulatory capacity [67]. MSCs express low-level MHC class II molecules and no co-stimulatory molecules such as CD80 and CD86 required for effector T cell induction to ensure allogeneic application [68]. The research related to the application of MSCs in bone and tooth regeneration is currently a hot topic in the field of tissue engineering [63, 69].

**Fig. 2:**
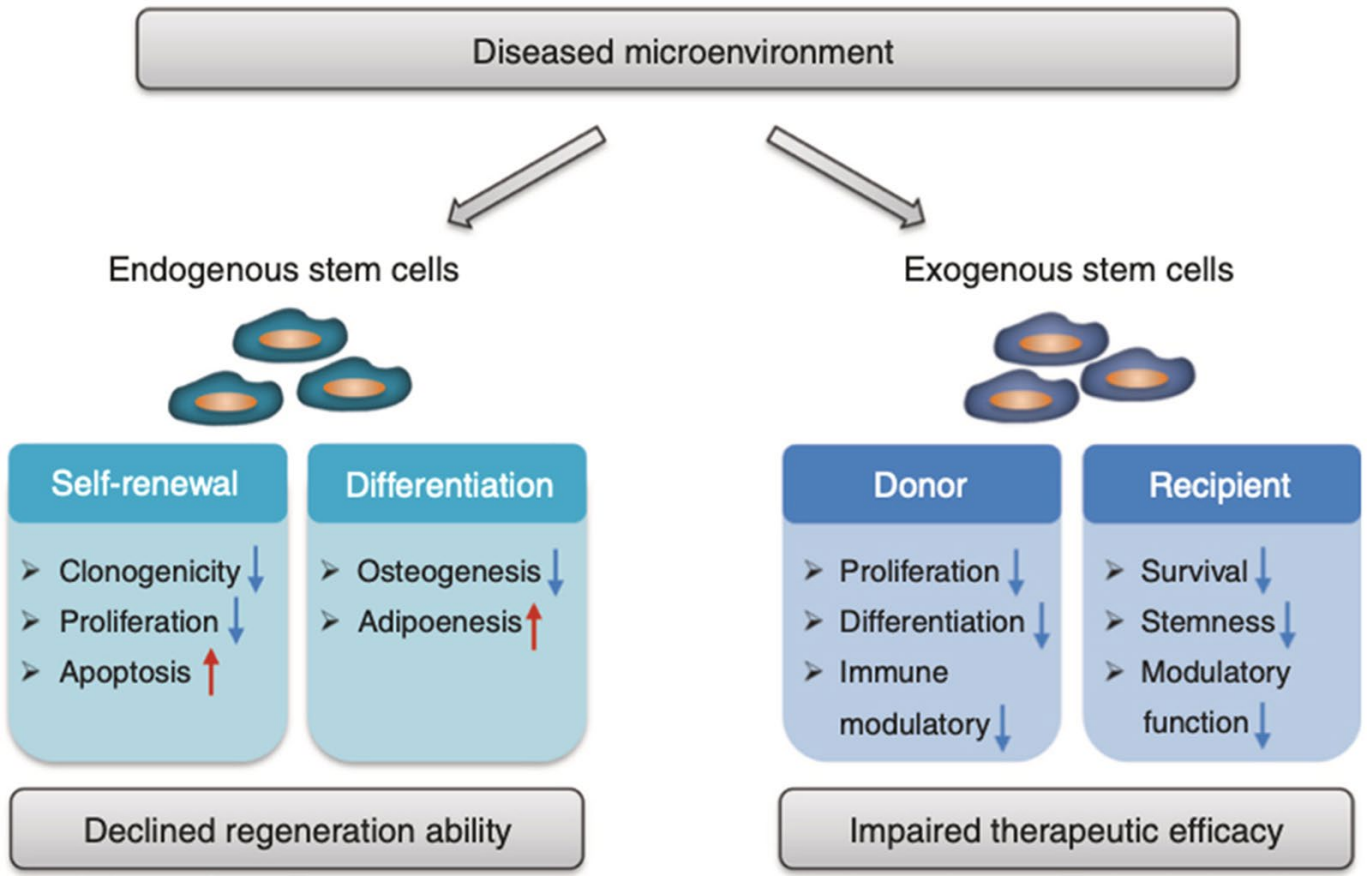
Microenvironment affects the stem cell-based tissue regeneration. The diseased microenvironment impairs functions of endogenous and exogenous stem cells leading to declined self-renewal ability and disturbed differentiation potential [63] Reprinted with permission. Copyright (2019), Springer Nature

For exogenous stem cells therapies, various techniques have been developed to achieve periodontal tooth-supportive tissue regeneration. Two review articles by Park CH et al. and Xu et al. had well summarized recent advances on exogenous stem cell-based therapies for periodontal tissue regeneration [19, 70]. However, exogenous stem therapy requires a large number of cells and high technical expertise, which increases the cost of treatment. In addition, there are some risk factors in the use of stem cell therapy, such as immune reaction, disease transmission, stem cells survival, cancer risk, etc. More detail on stem cell-treatment-associated risk factors could be found in a review article by Herberts et al. [71]. Nevertheless, the efficacy of stem cell therapy is not always fulfilled according to the microenvironment. The efficacy of transplanted exogenous stem cells is compromised by diseased microenvironment of the donors and the recipients (Fig. 2). On the other hand, the self-renewal and differentiation ability of endogenous stem cells are reduced in the diseased microenvironment that leads to compromised tissue regeneration [63, 72, 73]. Therefore, use of stem cell-CM could be a better alternative to direct use stem cells for periodontal regeneration that gives similar results to stem cells but eliminate the risks associated with the direct use of stem cells.

## Conditioned medium from MSCs (MSC-CM) as cell-free therapeutic strategy

**Fig. 3:**
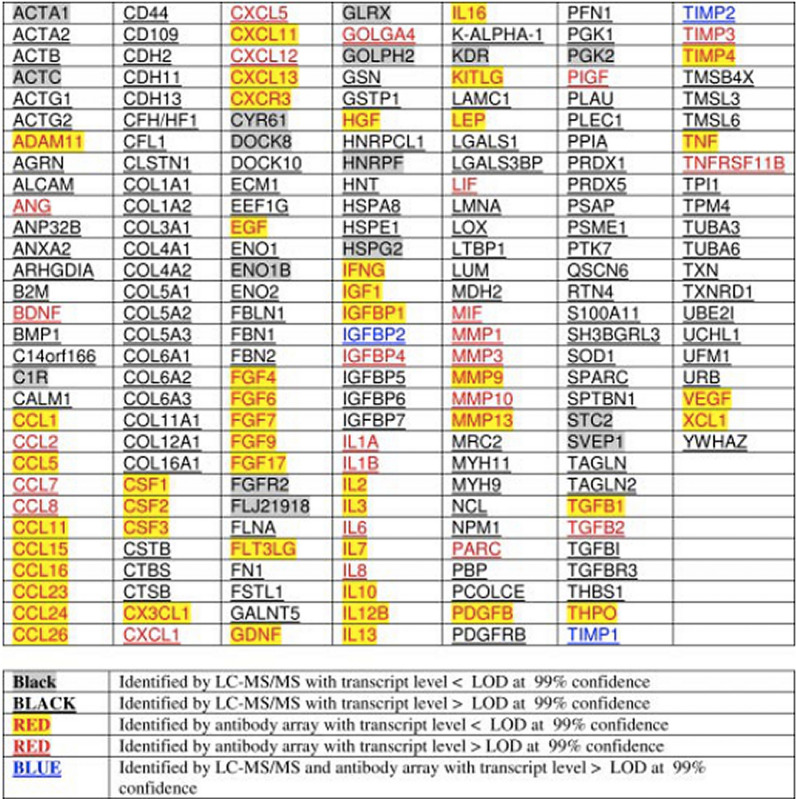
Unique gene products of MSC-CM identified by LC–MS/MS and antibody array [75] Reprinted with permission. Copyright (2007), American Society for Biochemistry and Molecular Biology

The function of MSCs appears not to be mediated through engraftment in the injured tissues but a ‘hit and run’ mechanism, which indicated that MSCs mainly act through the bio-active factors [25, 74]. The MSC-CM is a cocktail mixture of several hundred to thousands of different proteins, cytokines, growth factors, and enzymes. MSC-CM also contains extracellular vesicles (EVs) as a cargo of various proteins, coding and non-coding RNA, small RNAs, autophagosomes, mitophagosomes. EVs could be subdivided into apoptotic bodies, microparticles and exosomes [28]. Cytokine antibody array analysis revealed 201 unique gene products in human embryonic stem cell-derived MSC-CM (hESC-MSC-CM) (Fig. 3). These growth factors significantly drive the biological processes of metabolism, defense response, and tissue regeneration [75]. Shreds of literature had reported the concentration of different cytokines and growth factors in different MSC-derived CM. Some researchers have even proposed the possible role of certain growth factors or cytokines present in MSC-CM in tissue regeneration [24, 28, 75]. However, it is very difficult to claim the role of only a few growth factors or cytokines present in MSC-CM on tissue regeneration. All these cellular and biological products might play a role to give the cumulative results of tissue regeneration. Compared to cell-based therapies, CM may provide several advantages: (1) CM uses proteins rather than the whole cells to promote regeneration; (2) CM could be stored for a long time without using any toxic reagent such as DMSO; (3) The preparation of CM is more economical and CM can be mass produced; (4) The safety and efficacy evaluation of CM will be simpler, similar to traditional pharmaceutical preparations [28, 76].

## CM from different MSCs for periodontal tissue regeneration

**Fig. 4:**
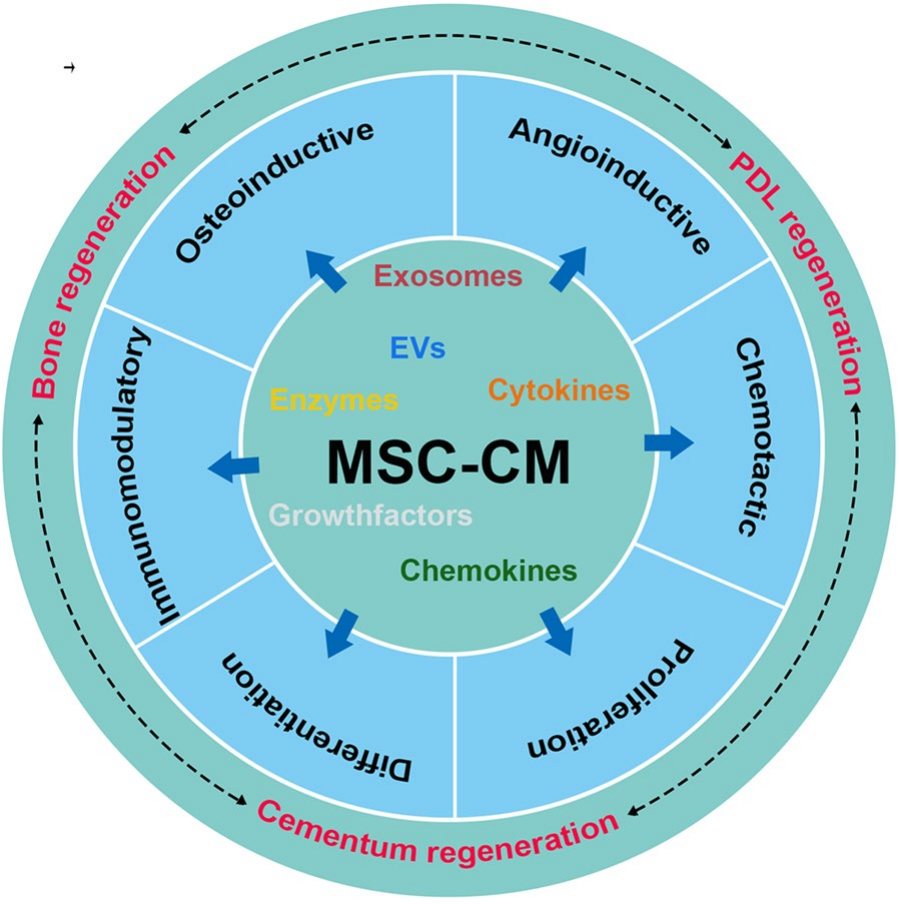
Biological activities of MSC-CM that could facilitate periodontal regeneration

CM from different sources of MSCs has been identified to have beneficial effects on the recipient, such as anti-inflammatory, anti-scarring, and immunomodulatory [77]. In recent years, MSC-CM has been widely used in the field of tissue regeneration [78–80], and its application in periodontal tissue regeneration is also gradually increasing [81, 82]. Osteogenesis, angiogenesis, cementogenesis, periodontal ligament regeneration and inflammation alleviation are key events to address during periodontal tissue engineering. Reports from literature had unraveled the various biological activities of MSC-CM, including, osteoinductive, angioinductive, chemotactic, immunomodulatory, and cell growth and differentiation (Fig. 4). These entire biological activities of MSC-CM could facilitate the periodontal tissue regeneration.

### MSC-CM-based periodontal tissue regeneration

MSC-CM could promote the regeneration of periodontal tissues. It has been reported that after transplantation of MSC-CM for 4 weeks, periodontal ligament like structures were seen between regenerated cementum-like structure and bone [82]. PDLSC-CM contains various growth factors, cytokines, extracellular matrix proteins, and angiogenic factors. Study has shown that PDLSC-CM promoted periodontal regeneration in a concentration-dependent manner [83]. 4 weeks after CM transplantation, histological images showed higher bone levels and newly formed periodontal tissues were observed in PDLSC-moderate and PDLSC-high groups compared with other groups. Collagen bundles, which bridged tooth root and alveolar bone, were evident in periodontal space of all sections. Periodontal ligament and gingiva are both important sources of stem cells. Gingiva is more accessible than periodontal ligament. PDLSC-CM and GMSC-CM had demonstrated a significant effect on periodontal regeneration by alleviating TNF-α and IL-1β expression and inducing BSP-II and Runx2 expression. Moreover, IL-10 expression was significantly higher in the GMSC-CM group than in the PDLSC-CM group and the control groups [84]. PDLSCs and GMSCs co‐cultured with APTG‐CM could form cementum and PDL‐like structures [85]. The exosomes of ASCs has been reported to be used as adjunctive therapy to nonsurgical periodontal treatment, and organized proliferating periodontal ligament tissue could be seen in interdental periodontal ligament space [86]. It has been reported that CM from osteogenically induced human maxilla BMSCs for 15 days promoted osteogenesis of hPDLSCs, and produced cementum-like mineralized and PDL-like collagen fibers [87].

Apical tooth germ cell-CM (APTG-CM) had been reported to promote cementogenic differentiation of PDLSCs [48]. Another study has shown that dental follicle cell-CM could induce cementogenic differentiation of rat ASCs, in which Wnt/β-catenin signaling pathway played a key role [88]. Odontoblast-CM has been reported to promote cementogenesis, which indicated the secreted products of odontoblasts could induce cementoblast differentiation [89]. Endogenous factors secreted by ASCs promote cementogenic differentiation of hPDLSCs [90]. MSC-CM had shown robust regeneration potential of alveolar bone and cementum in dog [91], indicating the possible application of MSC-CM on cementum regeneration.

Periodontal tissue regenerative potential of MSC-CM is mainly mediated by the cooperative effects of the cocktail of cytokines, growth factors, and enzymes such as insulin-like growth factor-1 (IGF-1), vascular endothelial growth factor (VEGF), transforming growth factor-β1 (TGF-β1) [92, 93], etc. Recent advances in MSC-CM-derived EVs including exosome-based periodontal tissue regeneration are well-reviewed in literature [94, 95]. Sakaguchi et al. prepared the cytokine cocktail (CC) by mixing insulin-like growth factor-1 (IGF-1), vascular endothelial growth factor-A (VEGF-A) and transforming growth factor-β1 (TGF-β1) to mimic MSC-CM secretomes. After 8 weeks, the regenerative periodontal tissues showed greater osteogenesis and cementogenesis by CC than enamel matrix derivative (EMD) [81]. IGFBP6 present in ASCs-CM had been reported to promote periodontal regeneration [90].

### The possible mechanism for MSC-CM-based periodontal tissue regeneration

#### MSC-CM-based angiogenesis

Studies have shown that the process of bone formation and tooth regeneration is coupled to angiogenesis [96, 97]. Osteogenesis-angiogenesis coupling are crucial for bone regeneration [98, 99]. Type H vessels are specific types of blood vessels that promote osteogenesis-angiogenesis coupling and bone regeneration [100]. MSC-CM is effective in the early phase of bone regeneration and angiogenesis in rabbit maxillary synovial floor elevation [101]. This study suggests that early vascularization facilitates the proliferation and migration of osteoprogenitor cells. MSC-CM increases angiogenesis via promoting migration and proliferation of endothelial cells [102]. VEGF [103] and FGF-2[104] in MSC-CM are proposed to be the main signaling factors that induce bone regeneration by promoting angiogenesis. However, the ability to promote angiogenesis could be relative to the type of stem cells. The proangiogenic potential of BMSC-CM is higher than DPSC-CM or even BMSC-derived EVs [97]. hBMSC-CM had been reported to promote matrigel tube formation and migration of human-derived lymphatic endothelial cells (HDLECs) [105]. Abundant numbers of literature had reported the angiogenic potential of MSC-CM [103, 106, 107]. The role of MSC-CM on type H vessel formation during bone regeneration has not been investigated yet. Future studies focusing on the role of MSC-CM in type H vessel formation are strongly recommended to further elucidate the mechanism of MSC-CM mediated osteogenesis-angiogenesis coupling and periodontal bone regeneration.

#### MSC-CM-based immunomodulation

Immune cells, including T cells, B cells, macrophages, and neutrophils play a vital role in the pathophysiology of periodontitis. Regulation of immune cells’ function to obtain the favorable immunomodulatory conditions for periodontal tissue regeneration is a challenging task. The immunomodulatory potential of MSC-CM can be utilized for periodontal tissue regeneration in clinics. MSC-CM had been reported to treat colitis by upregulating TGF-β, IL-10 and percentage of Treg cells, and downregulating IL-17 [108]. MSC-CM inhibits M0 macrophage apoptosis and induces M1 macrophage apoptosis. However, MSC-CM had no significant effect on macrophage proliferation and the expression of TNF-α and IL-10 [109]. M2 macrophages had anti-inflammatory properties that induce bone regeneration via the release of IL4, IL-10, and TGF- β). MSC-CM induces macrophage M2 polarization via NF-κB and STAT3 pathways [110]. Similarly, PDLSC-CM had shown M2 macrophage polarization potential by downregulating TNF-α and upregulating IL-10, Arg-1, and CD163 [111].

MSC-CM had been reported to induce neutrophil apoptosis in inflammatory conditions [112]. Human ASC-CM had shown potential to suppress inflammatory bone loss in the LPS-induced murine model [113]. MSC-CM increases the percentage of regulatory T (Treg) cells. Increased number of Treg cells alleviate periodontitis and induce periodontal bone regeneration [114]. MSCs cultured in hypoxic condition or in presence of anti-inflammatory agents such as IL-4 or IL-10 has shown better immunomodulatory properties [115, 116]. Therefore, MSC-CM obtained from optimized in vitro culture of MSC with improved immunomodulatory potential could be beneficial for periodontal tissue regeneration.

#### MSC-CM-based chemotaxis

The cytokines and growth factors in CM also play a key role in the chemotaxis of endogenous precursor cells. Chemotaxis of osteogenic and angiogenic precursor cells is essential for effective periodontal regeneration. It has been reported that MSC-CM could stimulate migration and proliferation of dog PDLSCs, which may enhance periodontal tissue regeneration [91]. MSC-CM could also promote the migration of endothelial cells and angiogenic differentiation [102, 117]. MSC-derived plasminogen activator inhibitor-1 (PAI-1) and tenascin-C significantly increase dermal fibroblast (DF) migration in vitro and improved wound healing in vivo by shortening the time for wound closure [118]. On the other hand, MSC-CM promotes macrophage chemotaxis via CCL2-CCR2 interaction [119]. MSC-CM induces higher chemotaxis of lymphatic endothelial cells (LEC) compared to VEGF-C and bFGF exogenous recombinant proteins [120].

MSCs could be harvested from different origins, such as bone marrow, adipose tissue, dental tissues, and umbilical cord. CM from different MSC sources have different effects on cells migration. BMSCs expresse highest mRNA levels of SDF1 and VCAM-1, and TNF-α. Priming of ASCs gained a significant increase in IDO1 and CCL5. And HUCMSCs release higher protein levels of IL-6, IL-8, MCP-1, ICAM1, HGF, MMP1 and CH3L1 [121]. MSC-CM (EVs-depleted) has higher chemotactic potential compared to MSC-EVs [97]. Therefore, the MSC-CM could be beneficial for endogenous precursor cells’ recruitment in the defect site during periodontal regeneration. The in vitro and in vivo effects of MSC-CM on bone regeneration, cementogenesis, angiogenesis, immunomodulation, and chemotaxis reported in literature are summarized in Tables 1 and 2, respectively.

**Table 1:** Periodontal regeneration-related in vitro biological activities of MSC-CM

S. No.	Source of MSC-CM	Cell type	Biological activity	Refs.
Bone regeneration				
1	hASCs	hPDLSCs	Upregulates osteoblastic gene expression in hPDLSCs	[90]
2	hBMSCs	hPDLSCs	Triggers osteogenesis of hPDLSCs	[87]
3	Healthy or inflamed PDLSCs	‘Inflamed’ PDLSCs	Healthy PDLSCs-CM rescues impaired-differentiation of inflamed-PDLSCs	[122]
Cementum regeneration				
1	hMSCs	Dog MSCs and dog PDLSCs	Promotes dog MSCs and dog PDLSCs proliferation and migration	[91]
2	rAPTGs	hGMSCs	Promotes differentiation of hGMSCs along the cementoblastic lineage	[85]
3	rDFCs	ASCs	Promotes ASCs towards cementoblast-like cells	[88]
4	rAPTGs	hPDLSCs	Promotes hPDLSCs towards cementoblast-like cells	[123]
Angiogenesis				
1	hMSCs	rMSCs	Increases angiogenesis	[82]
2	hMSCs	Human umbilical vein endothelial cells (HUVECs)	Promotes angiogenesis and migration of HUVECs	[103]
3	equine-PB-MSCs	ECs	Induces angiogenesis in equine vascular ECs	[106]
4	mMSCs and hEPCs	HUVECs	Promotes cell adhesion and proliferation	[107]
Immunomodulatory and anti-inflammatory				
1	hPDLSCs	RAW 264.7	Inhibits TNF-α expression	[83]
2	rPDLSCs	rBMDMs	Induces macrophage polarization towards the M2 phenotype	[111]
3	hPDLSCs	THP-1	Induces M1 macrophage polarization	[124]
Chemotaxis				
1	hMSCs	Dog BMSCs and dog PDLSCs	Enhances migration and proliferation of dMSCs and dPDLCs	[91]
2	hBMSCs	HUVECs	Promotes functional angiogenic effects	[97]
3	hMSCs and canine MSCs	ECs	Increases EC migration, proliferation and the formation of tubule-like structures	[102]
4	mMSCs	Dermal fibroblast	Induces dermal fibroblast migration	[118]
5	mMSCs	RAW264.7	Enhances the chemotaxis of RAW264 cells	[119]
6	hMSCs	Human dermal lymphatic ECs	Stimulates proliferation, migration, and tube formation of lymphatic ECs	[120]

**Table 2:** Summary of in vivo results showing the periodontal tissue regenerative potential of MSC-CM

Source of CM	Factors in CM	Study model	Route of delivery	Dose	Duration	Outcomes	Refs.
hPDLSCs	Matrix proteins, enzymes, growth factors, cytokines, and angiogenic factors	Rat periodontal defect	Fibrin coated collagen sponge	10 br	4 weeks	Enhances periodontal regeneration	[83]
hPDLSCs and hGMSCs	–	Rat periodontal defect	Collagen scaffolds		1, 2, and 4 weeks	Promotes periodontal regeneration	[84]
hMSCs	IGF-1, VEGF, TGF-1, and HGF	Rat periodontal defect	Collagen sponge	30 ll	2 and 4 weeks	Enhances periodontal regeneration via promoting osteogenesis and angiogenesis	[82]
hBMSCs	–	Ectopic transplantation in immunocompromised mice	Dentin block wrapped with hBMSC-CM-treated hPDLSC cell sheet	–	8 weeks	Promotes regeneration of cementum and PDL-like structure	[87]
rAPTGs	–	Ectopic transplantation in immunocompromised mice	PDLSCs (induced by APTG-CM) + CBB	–	6 weeks	Induces development of cementum and PDL-like structure	[48]
rAPTGs	–	Ectopic transplantation in immunocompromised mice	Cell sheet + dentin + CBB	–	8 weeks	Induces development of cementum and PDL-like structure	[85]
dMSCs	IGF-1, VEGF, TGF-β1, and HGF	Critical-size one-wall intrabony mandibular defects in dog	Atelo-collagen sponge	300 μL	4 weeks	Promotes alveolar bone and cementum regeneration	[91]
Cytokine cocktail-mimicking MSC-CM secretomes	IGF-1, VEGF-A, TGF-β1	Class II bifurcation premolar defect in dog	Hydroxypropyl cellulose	100 μL	8 weeks	Induces osteogenesis and cementogenesis	[81]
hMSCs	IGF-1, VEGF, TGF-β1, and HGF	Partially edentulous patients	MSC-CM + PLGA/β-TCP or MSC-C + ACS	3 mL	6 months	Promotes early bone formation and reduces inflammatory cell infiltration	[92]
hMSCs	IGF-1 VEGF TGF-b1	Rabbit bilateral maxillary sinus floor elevation model	β-TCP + MSC-CM	–	2, 4, 8 weeks	Promotes vascularization and early bone regeneration	[101]

## Summary and prospects

Recent studies have shown that stem cells are effective in tissue mainly via the paracrine effect [125]. The secreted molecules of stem cells play a key role in influencing the cross-talk communications between the cells and the surrounding tissues [29]. In this review, we summarized the regeneration of periodontal tissue by CM from different MSC sources, including BMSCs, PDLSCs, GMDCs, APTGs, DFGs, ADMPCs, ASCs, osteoblast, etc. Previous studies revealed that MSC-CM contains several cytokines, such as IGF-1, VEGF, TGF-β1, and HGF [82, 91, 126, 127]. These cytokines have been proved to regulate angiogenesis, cell migration, proliferation, and osteoblast differentiation to achieve the regeneration of periodontal tissue [127].

Although the applications of MSC-CM on periodontal regeneration have been proved useful in animal models from pre-clinical studies, much work needs to be done to apply it to clinics. The content of MSC-CM varies from cell type to culture condition and batch. It is impossible to get the MSC-CM containing similar secretomes in each treatment in clinics. Therefore MSC-CM cannot guarantee a similar effect in every treatment. The regenerative effect of MSC-CM is usually from the cumulative effect of a cocktail of cytokines and growth factors rather than a few factors present in elevated levels. Not having the worldwide consensus protocol for MSC-CM harvesting for tissue regeneration application is also one problem. So far, there is no data to illustrate that CM from which specific MSC origin is suitable for the specific tissue regeneration. This makes it difficult to choose the proper MSC origin for MSC-CM-based periodontal regeneration. Limited source and invasive procedures to harvest MSCs are key challenges of the use of autologous or allogenic MSC-CM for periodontal regeneration. CM from cell-sheet and co-culture of different cell types such as MSCs, ECs, monocytes, etc. could be more effective for periodontal regeneration compared to 2D expanded MSC-CM. Further in vitro, preclinical, and clinical studies are indispensable to improve the clinical efficacy of MSC-CM-based periodontal tissue regeneration.

## Conclusion

The role of stem cells in promoting tissue regeneration mainly depends on their paracrine function. The use of MSC-CM is safer and effective for periodontal tissue regeneration compared to MSC transplantation. The MSC-CM can be tailored as required using different drugs or culture conditions during in vitro culture of MSC. Moreover, the concentration of effective components and growth factors in MSC-CM can be optimized as required. MSC-CM-based periodontal tissue regeneration has the potential to eliminate the use of autologous and allogeneic stem cells. Based on the aforementioned facts, MSC-CM-based periodontal tissue regeneration has tremendous potential for bench-to-bed translation.

## Data Availability

Not applicable.

## Abbreviations

MSCs: Mesenchymal stem cells
CM: Conditioned medium
BOP: Bleeding on probing
PDLSCs: Periodontal ligament stem cells
DPSCs: Dental pulp stem cells
BMSCs: Bone marrow MSCs
MNPs: Mononuclear phagocytes
APCs: Antigen-presenting cells
PDL: Periodontal ligaments
AB: Alveolar bone
HSCs: Hematopoietic stem cells
PSCs: Periosteal stem cells
ASCs: Adipose-derived mesenchymal stem cells
DSCs: Dental tissue-derived stem cells
GFSCs: Gingival fibroblastic stem cells
DFSCs: Dental follicle stem cells
SHEDs: Stem cells from human exfoliated deciduous teeth
SCAP: Stem cells from the apical papilla
EVs: Extracellular vesicles
APTG: Apical tooth germ cell-CM
HDLECs: Human-derived lymphatic endothelial cells
DF: Dermal fibroblast
LEC: Lymphatic endothelial cells
HUVECs: Human umbilical vein endothelial cells
